# Information-Processing Entropy and Heterogeneous Sentiment Reaction Windows: Evidence from S&P 500 Stocks

**DOI:** 10.3390/e27121234

**Published:** 2025-12-05

**Authors:** Chi-Yao Peng

**Affiliations:** Department of Information Management, National Sun Yat-sen University, Kaohsiung 80424, Taiwan; d114020004@student.nsysu.edu.tw

**Keywords:** informational entropy, sentiment analysis, temporal heterogeneity, investor behavior, genetic algorithm, FinBERT, Bollinger Bands

## Abstract

This study examines the heterogeneous timing of market responses to financial news and its implications for informational uncertainty in price-adjustment dynamics. Empirically, stocks do not incorporate positive and negative sentiment at the same speed; instead, they exhibit asset-specific delays that stem from differences in investor attention, cognitive processing, and microstructural constraints. These unequal reaction windows increase the entropy of the information-transmission process, as sentiment shocks diffuse across assets in a dispersed and temporally misaligned manner. To quantify this heterogeneity, we develop a framework that integrates FinBERT-based sentiment classification, Bollinger Bands signal identification, and a Genetic Algorithm (GA) to estimate stock-specific sentiment reaction windows. Using S&P 500 data from 2021 to 2024, with 2022 to 2024 reserved for out-of-sample validation, the results show that GA-derived windows capture actual price-adjustment lags more accurately and significantly improve trading performance compared with fixed-window and technical-only benchmarks. In particular, incorporating news headline sentiment into the Bollinger Bands framework increases the win rate by approximately 5% over the testing period and leads to a significant improvement in overall returns. These findings demonstrate that the assimilation of sentiment is a time-dependent and non-uniform process shaped by behavioral and structural factors, offering new evidence that informational entropy—arising from delayed and heterogeneous reactions—plays a meaningful role in market efficiency and return dynamics.

## 1. Introduction

Investor sentiment increasingly shapes financial markets, yet reaction times to news vary markedly across assets due to differences in liquidity, visibility, and investor attention [1]. These unequal reaction speeds introduce informational uncertainty into the price-adjustment process, effectively increasing the entropy of how news-derived signals diffuse across financial instruments.

Prior studies have long documented that negative news often triggers stronger and more asymmetric market responses than positive news. Early evidence from Clarke and Statman [2] shows that bearish signals exert disproportionate influence on price adjustments, while García [3] further demonstrates that the effect of negative sentiment becomes even more pronounced during market downturns. This asymmetric sensitivity is also consistent with a broad class of Asymmetric GARCH models [4], which reveal that negative shocks typically generate larger volatility responses than positive shocks. These findings imply that sentiment-induced information does not diffuse uniformly across markets and contributes to heterogeneous, state-dependent reaction delays.

Such temporal heterogeneity presents a critical challenge: fixed sentiment windows, still widely used in practice, often misalign sentiment signals with actual price dynamics, thereby reducing predictive validity and degrading trading performance—even in advanced sentiment-driven models [1,5]. Building on Tetlock [6]’s behavioral and cognitive finance foundations, this study conceptualizes sentiment window heterogeneity as the asset-specific delay between sentiment release and market impact, reflecting dispersed and non-uniform information-processing behaviors among investors. Moreover, different stocks exhibit unequal reaction delays between the release of sentiment information and the subsequent price response, highlighting cross-sectional heterogeneity in sentiment adjustment speeds. To address this, we develop a robust trading framework that integrates a GA to fine-tune individualized sentiment reaction windows, enabling closer alignment between sentiment signals and Bollinger Bands–based technical indicators for improved execution precision. Accordingly, the central research questions are formulated as follows:

- RQ1. Do the sentiment reaction windows optimized by the GA reveal systematic temporal heterogeneity across stocks, reflecting asset-specific patterns in the entropy and dispersion of sentiment assimilation?
- RQ2. Can GA-optimized sentiment reaction windows reduce informational uncertainty and improve the alignment between sentiment shocks and price-adjustment dynamics—thereby enhancing the performance of Bollinger Bands—based trading strategies relative to fixed-window or sentiment-agnostic models?

An integrative theoretical perspective informs our research approach. Drawing from Behavioral Finance Theory [7,8], we consider how cognitive biases and limited attention shape delayed or asymmetric sentiment responses. Complemented by Information Processing Theory [9,10], the study emphasizes variability in how information is absorbed, filtered, and acted upon by different market participants—variability that contributes to temporal uncertainty and market-level informational entropy. Additionally, Signaling Theory [11] situates sentiment as an external informational cue interpreted differently across assets. Together, these perspectives provide a coherent foundation for linking heterogeneous sentiment assimilation to adaptive trading performance, offering both theoretical depth and practical relevance.

## 2. Literature Review

This chapter reviews the literature along four major themes: (1) the theoretical foundation of market sentiment and price deviations, (2) the evolution of sentiment analysis techniques and language models for financial text, (3) the trading logic of the Bollinger Bands technical indicator, and (4) the application of genetic algorithms to trading parameter optimization. These discussions establish the theoretical basis for the framework and methodological design of this study.

### 2.1. Media Sentiment and Market Response

The Efficient Market Hypothesis (EMH) posits that asset prices fully reflect all available information. However, empirical evidence suggests that media sentiment and investor reactions often cause short-term price deviations. Tetlock [6] proposed a quantitative framework for extracting tonal shifts in financial news and constructed a “media sentiment index”, demonstrating its significant predictive power for short-term stock returns, particularly the strong association between negative tone and subsequent price declines. Building on this, García [3] further showed that the impact of negative news is more pronounced during bear markets, underscoring the amplifying effect of sentiment on market volatility.

### 2.2. Applications of FinBERT in Financial Sentiment and Forecasting Research

FinBERT is a language model trained explicitly on financial text and is noted for its strong performance in sentiment classification within financial contexts. Zhou et al. [12] employed sentiment indicators to examine the diffusion of investor sentiment at the individual stock level and found that emotions captured by text-based models propagate across different stocks, thereby influencing cross-sectional returns. Sahu et al. [13] utilized FinBERT to extract managerial sentiment from the Management Discussion and Analysis (MD&A) sections of corporate annual reports. Their findings indicate that, when macroeconomic uncertainty rises, a more positive managerial tone can mitigate the adverse impact of uncertainty on stock liquidity, suggesting that sentiment features are increasingly recognized as critical measures of information asymmetry and predictors of market reactions. Zhang et al. [14] combined Chinese research report texts with firm fundamentals to propose a multi-module fusion model, MFF FinBERT, which achieved an accuracy rate of approximately 79% in forecasting one-year stock price trends, significantly outperforming traditional models. Overall, FinBERT not only delivers accurate sentiment classification in financial news and corporate disclosures but is also increasingly employed to investigate how sentiment diffusion shapes asset pricing, to analyze market behavior under macroeconomic uncertainty, and to enhance long-horizon stock price forecasts by integrating textual and fundamental data. These findings underscore the superior capability of FinBERT to extract sentiment signals from financial texts, making it an indispensable tool in both financial market research and practical applications.

### 2.3. Technical Analysis and Bollinger Bands Strategy

**Figure 1:**
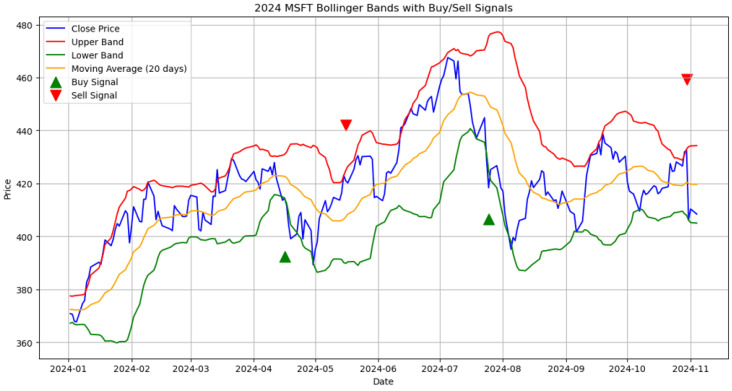
Bollinger Bands with buy and sell signals for Microsoft (MSFT) in 2024.

Bollinger Bands are a commonly used technical analysis strategy that integrates a moving average with two accompanying bands set at two standard deviations above and below the average, delineating a reasonable range of price fluctuations and identifying potential overbought or oversold conditions. Ni et al. [15], using the constituents of the Taiwan 50 Index as their research sample, conducted an empirical analysis and found that when stock prices touch the lower bound of the Bollinger Bands, investors adopting a buy-and-hold strategy often achieve significantly positive abnormal returns. These findings suggest that Bollinger Bands not only provide signals to help identify market conditions but also offer valuable guidance for formulating investment strategies and determining optimal market entry and exit points. Figure 1 illustrates a typical Bollinger Bands pattern for Microsoft (MSFT) in 2024, showing upper and lower bands, a 20-day moving average, and corresponding buy and sell signals.

### 2.4. Application of GA in Trading Strategy Optimization

GA have demonstrated considerable potential for applications in technical analysis and the optimization of trading strategies within financial markets. Gao et al. [16] proposed a hybrid approach that takes news sentiment indices and market data as inputs, integrating recurrent neural networks (RNNs) with evidential reasoning rules, while employing a GA to search for the optimal combination of weights and hyperparameters. Empirical results indicate that this method significantly improves prediction accuracy for the price movements of the S&P 500, Dow Jones Industrial Average, and NASDAQ 100, which in turn enhances cumulative returns and risk-adjusted performance. Similarly, Gülmez [17] incorporated news sentiment, along with multiple technical indicators, into a deep learning framework, confirming that the fusion of sentiment signals and technical features can further enhance the accuracy of stock trend forecasting. Collectively, these studies highlight that strategy optimization using GA can effectively strengthen the robustness of market trend prediction models and contribute to improved investment performance.

## 3. Methodology

### 3.1. Data and Sentiment Analysis

This study uses S&P 500 constituent trading data from Yahoo Finance (2021–2024), including daily open, close, high, low prices, and volume, alongside headlines and subheadlines from The Wall Street Journal. Prior research indicates that news headlines often contain the most informative elements of a news article, making it possible to extract sentiment effectively from headlines alone. For example, Anamika and Subramaniam [18] construct sentiment indices exclusively from news headlines and find that headline sentiment significantly affects cryptocurrency returns, confirming its validity as a market-relevant signal. Similarly, Liu et al. [19] adopt a headline-only sentiment approach to examine financial networks and show that headlines sufficiently capture the core tone and market-moving information embedded in financial news. Taken together, these recent studies suggest that headlines provide a cleaner and higher-signal textual source than full news articles, which often contain substantial noise, making headline-based sentiment particularly suitable for NLP-driven financial analysis. Building on the above literature, employing news headlines as the source for sentiment analysis in this study is empirically supported and methodologically sound. To further enhance sentiment identification, subheadlines are also incorporated. We use the FinBERT model from Hugging Face to classify each headline and subheadline as Positive, Negative, or Neutral, with GPU acceleration to improve computational efficiency.

### 3.2. Model Selection and Data Preprocessing

News sentiment classification is performed using the FinBERT model (yiyanghkust/ finbert-tone) available on the Hugging Face platform. This model classifies financial texts into three sentiment categories: Positive, Negative, or Neutral. To enhance inference efficiency, the model is implemented using GPU acceleration.

### 3.3. Sentiment Labeling Rules

Let

- $s_{h}\in \left\{-1,0,+1\right\}$ be the sentiment label of the headline
- $s_{s}\in \left\{-1,0,+1\right\}$ be the sentiment label of the subheadline,

where −1 = *Negative*, 0 = *Neutral*, and +1 = *Positive* sentiment, each independently assigned from FinBERT classification.

The final sentiment label y∈{Positive,Negative,Neutral} is determined by the directional agreement between the headline and subheadline sentiments, as follows:

$$y=\left\{\begin{array}{cc} \mathrm{Positive}, & \mathrm{if}\;s_{h}=+1\wedge s_{s}=+1\\ \mathrm{Negative}, & \mathrm{if}\;s_{h}=-1\wedge s_{s}=-1\\ \mathrm{Neutral}, & \mathrm{otherwise} \end{array}\right.$$

A non-neutral label is assigned only when headline and subheadline align; otherwise, disagreement defaults to Neutral, enhancing robustness. This label then guides trading signal timing and direction.

To illustrate the sentiment classification process, Table 1 presents two representative examples of news reports concerning Apple Inc. in late 2024. Each article’s headline and subheadline were independently evaluated using the FinBERT model, a domain-specific transformer pretrained on financial text corpora. For each textual segment, FinBERT produces a categorical sentiment label—*Positive*, *Neutral*, or *Negative*—along with a corresponding confidence score derived from the model’s softmax output probabilities.

Within the classification framework adopted in this study, a non-neutral label is assigned only when the headline and subheadline exhibit consistent sentiment polarity. In cases of divergence, the overall sentiment defaults to *Neutral*. The resulting final sentiment label is then used as an exogenous input to the trading framework to determine both the directional bias (buy or sell tendency) and timing adjustments in the generation of sentiment-enhanced trading signals.

### 3.4. Technical Indicators and Signal Generation

The proposed strategy employs the Bollinger Bands (BB) as the core technical indicator to identify potential price reversals under varying market volatility conditions. Following Bollinger [20], the bands are constructed as a dynamic envelope around the moving average, capturing the dispersion of prices relative to their recent mean:

(1) $${\mathrm{Upper}}_{t}=MA_{t}+2\sigma_{t},\;{\mathrm{Lower}}_{t}=MA_{t}-2\sigma_{t}$$

where $MA_{t}$ denotes the 20-day moving average of closing prices, and $\sigma_{t}$ represents the corresponding rolling standard deviation. The constant multiplier of 2 serves as a volatility buffer, encompassing approximately 95% of price observations under the assumption of near-normal returns.

A *buy* signal is generated when the market price falls below the lower band, indicating potential oversold conditions and mean-reversion opportunities, whereas a *sell* signal is triggered when the price exceeds the upper band, suggesting temporary overbought momentum. This rule-based mechanism enables the strategy to adaptively respond to shifts in volatility, providing a systematic framework for identifying reversals while filtering out transient noise.

When integrated with sentiment analysis, the BB signals are further refined by psychological market states, allowing the timing and direction of trades to adjust dynamically according to news-derived sentiment conditions.

### 3.5. Experimental Design

To investigate the impact of delayed buy and sell signals caused by sentiment windows on strategy performance, this study first used 2021 stock and news sentiment data to train the GA and determine the optimal sentiment window parameters for each stock. The GA-optimized parameters were then applied to Bollinger Bands–based trading strategies over the 2022–2024 period.

**Figure 2:**
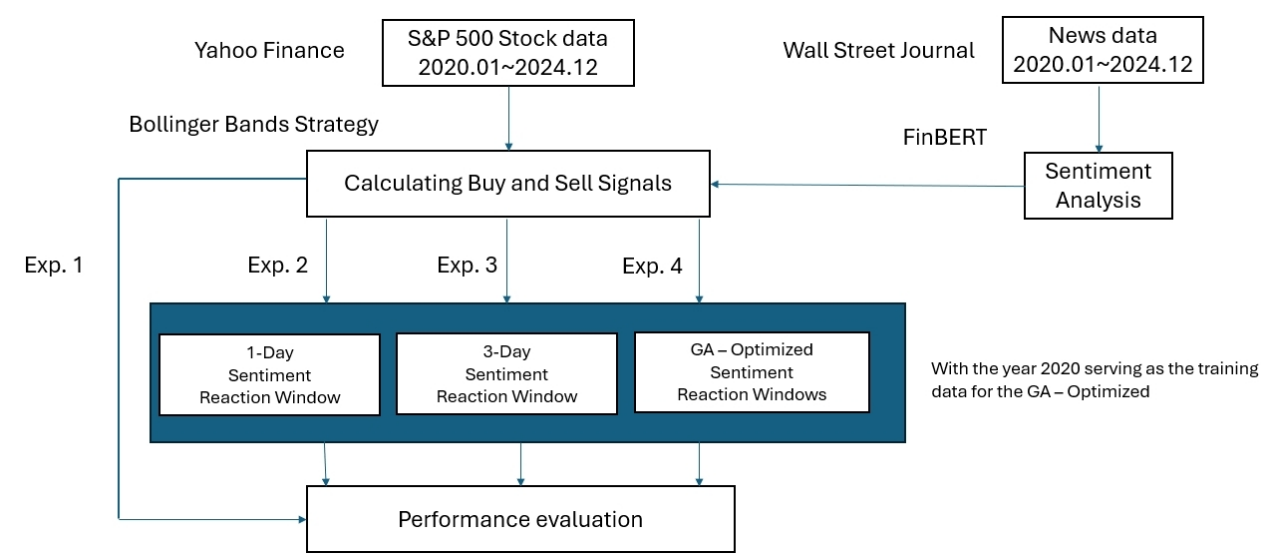
Research framework integrating FinBERT-based sentiment analysis, Bollinger Bands strategy, and GA optimization. The year 2021 serves as the training data for the GA.

Figure 2 presents the overall research framework. Financial data were obtained from *Yahoo Finance* and news data from the *Wall Street Journal*. FinBERT was employed to classify news sentiment, while the Bollinger Bands strategy generated buy and sell signals based on stock price dynamics. Four experimental setups (Exp. 1–Exp. 4) were designed to evaluate the influence of different sentiment window configurations on trading performance, as shown in Table 2.

Let *d* denote the sentiment windows (in days). Under the sentiment–adjusted condition, the signal modification logic is defined as follows:

- If a buy signal is generated on day *t*, and the sentiment observed on day *t* − *d* is **Negative**, the signal is blocked.
- Conversely, if a sell signal is generated on day *t*, and the sentiment on day *t* − *d* is **Positive**, the signal is suppressed.

### 3.6. GA Design

The robustness of the GA employed in this study is well supported by prior research. Fleming and Purshouse [21] characterize evolutionary algorithms as robust search methods capable of handling multimodality, discontinuity, randomness, and noise. More recent evidence corroborates this inherent stability: GA-based approaches remain effective in high-dimensional and noisy environments [22], and optimized GA variants maintain reliable performance even under substantial measurement variability [23]. Building on this foundation, the GA in our framework optimizes the sentiment-reaction windows, where each chromosome encodes the positive and negative sentiment delays as follows:

(2) $${\mathrm{Chromosome}}_{i}=\left[d_{\mathrm{pos}},d_{\mathrm{neg}}\right]$$

Here, $d_{\mathrm{pos}}$ represents the delay (in days) of positive sentiment affecting sell signals, while $d_{\mathrm{neg}}$ denotes the delay of negative sentiment affecting buy signals. The gene values are defined as integers ranging from 0 to 14. The parameters used for the GA are summarized in Table 3. At the end of each generation, a callback function is invoked to record the best fitness value, which is used to monitor the convergence of the algorithm.

### 3.7. Fitness Function Design: Maximizing Total Return

The fitness function in the GA is designed to maximize the total return, defined as:

(3) $$\mathrm{Total}\;\mathrm{Return}=\prod_{t=1}^{T}\left(1+R_{t}\right)-1,$$

where $R_{t}$ denotes the return of an individual trade at time *t*, computed as the percentage change between the buy and sell prices of that trade.

The total return formula reflects compounding for accurate capital growth measurement. Parameter sets with no trades receive zero return to avoid bias. Paired-sample *t*-tests compare Exp. 4’s returns with other groups to assess whether GA-optimized sentiment windows yield significant gains over non-optimized or fixed-window models.

## 4. Empirical Results and Analysis

**Figure 3:**
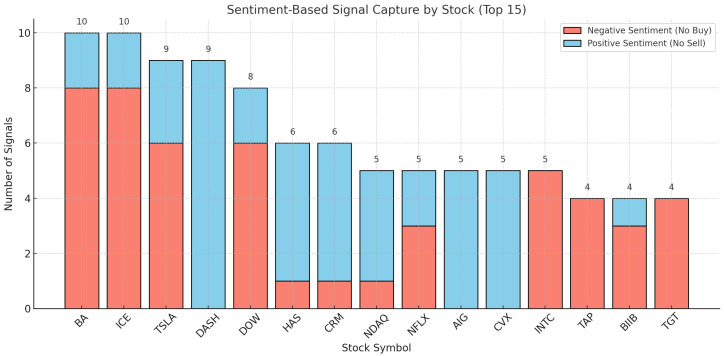
Sentiment-Based Signal Capture by Stock (Top 15) for 2022–2024. The figure shows the number of captured signals under negative and positive sentiment conditions, totaling 205 instances: 111 negative sentiment (No Buy) and 94 positive sentiment (No Sell) signals. These results confirm that the sentiment filter effectively constrained trading decisions and captured heterogeneous sentiment reactions across stocks.

The experimental results of the GA optimization are shown in Figure 3. As illustrated, the sentiment-based trading framework effectively captured shifts in market emotion during 2022–2024, identifying 111 *negative sentiment–no buy* events and 94 *positive sentiment–no sell* events, yielding a total of 205 sentiment-driven signals. These results indicate that the GA-optimized sentiment reaction windows dynamically suppressed trades under unfavorable psychological conditions while preserving positions during favorable sentiment states, thereby enhancing decision timing and improving the overall robustness of the trading strategy.

This section addresses two objectives from a diffusion-entropy perspective: (1) evaluating how sentiment reaction windows shape the temporal structure of trading performance, and (2) analyzing the heterogeneity in stock-level response delays, which contributes to the diffusion entropy of sentiment as it propagates across assets. A comparative assessment of the strategy groups is then presented.

### 4.1. Performance Metrics Comparison: Total Return and Win Rate

Table 4 reports average total return, win rate, return standard deviation, and *p*-values from paired-sample *t*-tests versus the baseline (Exp. 1) for 2022–2024. Sentiment-integrated strategies (Exp. 2–4) outperform the technical-only baseline, with Exp. 4 (GA-optimized) achieving the best performance.

The results clearly show that all sentiment integrated strategies (Exp. 2–Exp. 4) outperform the baseline technical-only strategy (Exp. 1) in terms of average total return and win rate. Among them, Exp. 4, which applies GA-optimized sentiment window parameters, achieves the highest performance across all metrics. The extremely low *p*-values from paired *t*-tests confirm that these improvements are statistically significant.

### 4.2. Quantifying Sentiment Response Heterogeneity via Coefficient of Variation

To validate that sentiment response timing varies across assets, we measure the dispersion of GA-optimized sentiment delay parameters using the coefficient of variation (CV). As shown in Table 5, CVs are 0.657 (positive) and 0.675 (negative), both exceeding 0.65, indicating wide dispersion. This supports the sentiment reaction heterogeneity hypothesis and justifies stock-specific rather than fixed sentiment windows calibration. The cross–sectional patterns of the GA–optimized sentiment windows further clarify the sources of these heterogeneous delays. Stocks with lower liquidity or larger bid ask spreads tend to show longer delays, reflecting the influence of market microstructure frictions on information incorporation. In contrast, assets with higher investor attention or greater market visibility generally exhibit shorter delays, indicating behavioral differences in attention allocation and information processing. Taken together, these empirical patterns suggest that both microstructural and behavioral factors contribute to the observed heterogeneity in sentiment reaction windows.

### 4.3. Sentiment Windows Clustering and Strategy Performance

**Figure 4:**
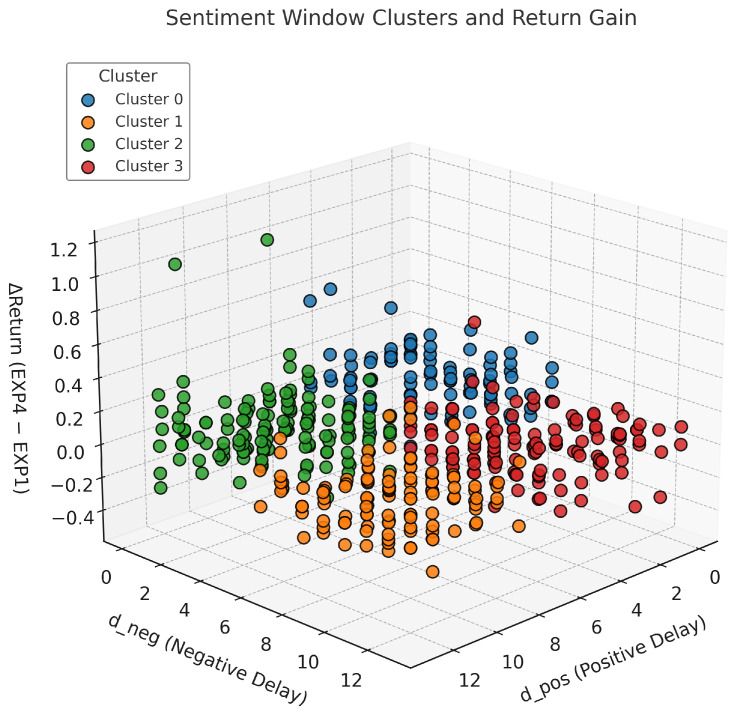
Asset distribution in the space of optimized sentiment windows ($d_{\mathrm{pos}}$, $d_{\mathrm{neg}}$) and return improvement (ΔReturn), clustered via K-Means.

To examine heterogeneity in sentiment reaction windows and their effect on performance, we visualize a three-dimensional space of each stock’s optimized positive lag ($d_{\mathrm{pos}}$), negative lag ($d_{\mathrm{neg}}$), and return improvement (Δ Return = EXP. 4 − EXP. 1) from the GA. Figure 4 plots all assets in this space, with points color coded according to K-Means clustering. Distinct groupings in $\left[d_{\mathrm{pos}},d_{\mathrm{neg}}\right]$ indicate structural differences in sentiment reaction timing, supporting the hypothesis that sentiment windows vary across financial instruments.

### 4.4. Empirical Findings and Research Validation

The experimental results yield three main findings:

1. Delayed Sentiment Effects: Exp. 2 and Exp. 3 show that news sentiment impacts prices with a measurable lag, not instantaneously.
2. Response Heterogeneity: Exp. 4’s superior results confirm that sentiment windows vary by asset, supporting stock-specific calibration.
3. GA Optimization Benefits: GA-optimized windows in Exp. 4 notably boost performance, proving GA’s practical value for trading parameter optimization.

## 5. Conclusions and Implications

### 5.1. Conclusions

This study investigates whether heterogeneous timing exists in the assimilation of news sentiment across different market assets [24]. To address this question, we develop a quantitative trading framework that integrates FinBERT–based sentiment extraction, Bollinger Bands technical indicators, and a GA to optimize sentiment reaction windows, consistent with prior research highlighting the effectiveness of GA-enhanced sentiment-driven prediction models [25]. The GA is applied to each stock individually to identify the optimal lag days that best capture delayed market responses to both positive and negative sentiment.

The empirical design evaluates four strategy groups (Exp. 1–Exp. 4), including a purely technical-indicator benchmark, a fixed sentiment window, and GA-optimized individualized sentiment windows. Using out-of-sample data from S&P 500 constituents between 2022 and 2024, the results reveal substantial cross-asset variation in sentiment reaction timing, displaying distinctive and asset-specific sentiment assimilation patterns. These findings directly confirm RQ1, demonstrating that heterogeneous sentiment reaction windows indeed exist across stocks.

Furthermore, strategies incorporating GA-optimized individualized sentiment windows consistently outperform both fixed-window and sentiment-agnostic benchmarks in terms of total return and win rate. This performance superiority shows that tailoring sentiment reaction windows enhances the adaptability of Bollinger Bands–based trading strategies and improves risk-adjusted outcomes. These results provide clear empirical support for RQ2, indicating that optimized sentiment windows materially strengthen trading performance.

Overall, the evidence confirms that (1) sentiment reaction timing is heterogeneous across assets and (2) GA-based optimization offers a systematic, effective mechanism for exploiting this heterogeneity to improve trading outcomes.

- Exp. 4 (GA-Optimized) achieves the highest total return and win rate, supporting the hypothesis that sentiment reactions exhibit temporal heterogeneity across assets.
- Paired-sample t-tests show that the performance of Exp. 4 is significantly different from all other strategy groups—including the purely technical benchmark—with statistical significance (*p* < 0.05).

### 5.2. Academic Implications

This study makes three key contributions to the academic literature on sentiment-based trading, market microstructure, and the temporal dynamics of information diffusion.

First, it proposes and empirically validates the hypothesis of sentiment window heterogeneity at the individual asset level. Unlike conventional models that assume uniform or instantaneous sentiment effects, this study introduces a novel GA-based procedure for identifying optimal sentiment reaction lags for each stock. This methodological advancement fills an important gap by showing that sentiment signals diffuse across assets with distinct temporal structures, revealing a form of cross-asset temporal heterogeneity comparable to diffusion entropy.

Second, the study develops a reproducible and extensible empirical framework that integrates FinBERT-based sentiment classification, Bollinger Bands technical indicators, and GA-driven metaheuristic optimization. This unified architecture enables large-scale examination of heterogeneous sentiment assimilation and provides a robust methodological foundation for future research on sentiment-augmented trading strategies and time-adaptive market models.

Third, the findings offer new insights into behavioral finance theories concerning limited attention, gradual information incorporation, and bounded rationality. The pronounced disparities in sentiment lag structures across assets demonstrate that markets do not internalize public information synchronously, a pattern consistent with entropy-based assessments of behavioral efficiency in financial markets [26]. This provides quantifiable evidence of behavioral and microstructural inefficiencies, while also highlighting the temporal dispersion and nonuniform diffusion of sentiment signals within financial markets.

### 5.3. Practical Implications

This study also provides valuable implications for financial practitioners, particularly those engaged in the development of sentiment-driven quantitative strategies. The empirical findings indicate that employing individualized sentiment reaction windows—rather than imposing a uniform lag structure across assets—can substantially enhance trading performance. This emphasizes the importance of incorporating asset-specific behavioral patterns into model design. Practitioners are therefore advised to move beyond overly simplified assumptions regarding sentiment response timing and instead adopt adaptive mechanisms that account for the unique characteristics of each security.

Moreover, the proposed framework—which integrates FinBERT for financial text analysis, Bollinger Bands for technical signal generation, and a GA for parameter optimization—forms a scalable and modular architecture. Its flexibility allows for seamless adaptation across asset classes, time horizons, and market regimes, making it highly suitable for deployment in real-world quantitative investment platforms and algorithmic trading systems.

### 5.4. Limitations and Future Research

While this study presents meaningful findings, several limitations should be acknowledged. First, the sentiment data are derived solely from headlines and subheadings of *The Wall Street Journal*, which may limit the breadth and representativeness of sentiment signals. Expanding the sentiment source to include alternative channels such as social media, analyst commentary, or earnings releases could yield a more comprehensive sentiment profile.

Second, the modeling of sentiment reaction lags is based on static integer values. In practice, the impact of sentiment on asset prices may decay gradually over time, implying that more flexible or continuous lag structures may better reflect market behavior.

Third, the study does not explore the relationship between sentiment lag parameters and firm-specific characteristics, such as market capitalization, sector classification, or liquidity. Understanding whether these fundamental attributes can predict optimal sentiment delays remains an important avenue for future investigation.

Finally, the current framework treats assets independently and does not account for potential cross-asset sentiment spillover effects. In reality, a single news event may influence multiple stocks simultaneously, particularly within the same sector or supply chain.

Future research may address these limitations by incorporating richer sentiment sources, investigating the drivers of sentiment-delay heterogeneity, and modeling inter-asset sentiment transmission. In addition, future extensions may explore whether the proposed framework generalizes across different markets, sectors, and languages that operate under distinct information environments, thereby assessing the robustness and external validity of heterogeneous sentiment reaction windows. These extensions would further enhance the robustness, interpretability, and real-world relevance of sentiment- based trading strategies.

## Figures and Tables

**Table 1:** Examples of News Sentiment Determination for Apple Inc.

Date	Article Title	Subtitle	Final Sent.
28 October 2024	Apple iPhone 16 Sales Banned in Indonesia Over Investment Issue	Other Apple products aren’t subject to the ban	**Negative**
31 October 2024	Apple Sales *Hit Quarterly Record* as iPhone Business Rebounds	The company’s smartphone, which accounts for half its revenue, saw renewed *growth* ahead of the release of Apple Intelligence this month	**Positive**

*Note*: Each article’s title and subtitle are analyzed separately using the FinBERT model, which assigns sentiment labels (Positive, Neutral, Negative) and associated confidence scores. *Source*: The Wall Street Journal.

**Table 2:** Sentiment Window Experiments.

Exp.	Description of Trading Rule
Exp. 1	Bollinger Band on day *t* (Baseline).
Exp. 2	Bollinger Band on day *t* plus sentiment indicator on day t−1.
Exp. 3	Bollinger Band on day *t* plus sentiment indicator on day t−3.
Exp. 4	Bollinger Band on day *t* plus sentiment indicator on the day prior to *t*, using a GA-optimized sentiment window for each stock.

**Table 3:** GA parameter settings.

Parameter	Value
Number of generations	30
Population size per generation	30
Number of parents selected for mating	3
Mutation probability	0.2
Crossover probability	0.8
Number of elite individuals retained	2
Stopping criterion	10

**Table 4:** Performance Comparison of Strategies (2022–2024).

Strategy	Avg Ret.	Win Rate	*p*-Value vs. Exp. 1
Exp. 1	0.184	68.79%	–
Exp. 2	0.227	73.56%	<0.001 ***
Exp. 3	0.228	73.56%	<0.001 ***
Exp. 4	0.229	73.96%	<0.001 ***

*Note:* *** *p* < 0.001.

**Table 5:** Coefficient of Variation for GA-Optimized Sentiment Windows.

Sentiment	Mean Window (Days)	Std. Dev.	CV
Positive	6.17	4.05	0.657
Negative	6.07	4.09	0.675

## Data Availability

The data in this study are available from the corresponding author upon reasonable request.
